# Morphology of Proximal Femur in South-West Coast of India

**DOI:** 10.5704/MOJ.2011.022

**Published:** 2020-11

**Authors:** SU Kamath, S Agarwal, J Austine

**Affiliations:** 1 Department of Orthopaedics, Manipal Academy of Higher Education, Manipal, India; 2 Department of Orthopaedics, Sumit Nursing Home, Meerut, India; 3 Department of Orthopaedic Surgery, Jawaharlal Institute of Post Graduate Medical Education and Research(JIPMER), Pondicherry, India

**Keywords:** proximal femur, morphometry, south Indian, hip, arthroplasty

## Abstract

**Introduction:**

With a higher proportion of young individuals undergoing uncemented hip arthroplasty, a close match in the dimension of the proximal femur and the implanted prosthesis is paramount. This is a study to gain insight into geographical variation in proximal femur morphology to determine the reference values to design uncemented femoral stems for a south Indian population, and also the effect of ageing and gender on the proximal femur morphology.

**Materials and Methods:**

The study comprised of two groups. For the first group, 50 unpaired dry femur bones were obtained from adult human cadavers; and the second group was a clinical group of 50 adult patients. Standardised radiographic techniques were used to measure the extra-cortical and intra-cortical morphometric parameters. Based on these, dimensionless ratios were calculated to express the shape of the proximal femur. The data were expressed in terms of mean and standard deviation and a comparison made with other studies.

**Results:**

A significant difference was noted across various population subsets within the Indian subcontinent and also in comparison to the Western population, suggestive of regional variation. The measurements made in cadaveric bone differed significantly from those in live patients, especially the femoral head diameter and extra-cortical and intra-cortical width. Femoral offset, head height and diameter were significantly less in females.

**Conclusion:**

The south Indian population needs customised implants with an increase in neck shaft angle and a decrease in intra-cortical and extra-cortical width for press fit in hip arthroplasty. The variation between the two sexes must also be accounted for during prosthesis design.

## Introduction

Orthopaedic literature is replete with exemplifications hailing total hip arthroplasty (THA) as one of the most successful and cost effective procedures in medicine^1^. Since THA has now become inherent with mainstream orthopaedic practice, we are now in an era where our goal is to achieve ideal post-operative functional outcomes. This has gained further importance due to the higher proportion of young individuals undergoing hip replacement^2,3^.

Interest has been renewed in the development of non-cemented arthroplasty as an alternative to fixation with cement. The prime reason behind the development of uncemented prosthesis was the complication of aseptic loosening. The other reason was the patient age groups below 65 years, which necessitated leaving behind better bone stock for revision arthroplasty.

Reports of the cemented prosthesis in the younger patients less than 50 years have not been satisfactory. Dorr *et al* reviewed 81 cemented total hip arthroplasties in patients with age ranging from 14 to 45 years and found that after two to five years of the procedure satisfactory results were seen in 78% which dropped to 72% after five years. Poorest results were seen in those less than 30 years of age^4^.

One approach to non-cemented stem design is based on the proposition that a stem shape that closely resembles the anatomy of the femur, particularly in the proximal region, can achieve intimate contact and stability, and approximate the stress and strain pattern of the normal femur^5^. Some anatomic studies suggest that exact total fit of a non-cemented prosthesis to the cortical envelope is not a realistic goal because of the large variation in anatomy and age-related changes in geometry^6-8^. However, the fit achieved with an anatomic design, emphasising the maximum fit in priority areas of contact, should result in maximal load transfer to cortical bone and resist not only axial and bending loads but the important torsional loads as well^5,9^. A critical factor for success must be the health of the trabecular and cortical envelope into which the prosthesis is delivered^10^.

Cadaveric studies have shown that age and gender have a bearing on the proximal femur morphology. It has been noted that cementless femoral prostheses of one standard shape cannot provide a close fit to the endosteal contours especially in the young and the elderly women^7,11^. Mahaisavariya *et al* utilised a three-dimensional reverse engineering technique to analyse cadaveric femora in a Thai population and observed variations in the femoral head diameter, neck anteversion angle and the femoral canal in comparison to a Caucasian population^12^. An anthropometric study conducted by Siwach *et al* observed that the proximal femur morphology differed markedly amongst various ethnic groups to the extent that certain standard prosthesis would not be conducive for implantation in certain subsets of the Indian femora^13^.

The aim of our study was to determine the reference values to design non-cemented femoral stems for a south Indian population and to study the effect of ageing and gender on morphological parameters of the proximal femur in a clinical group.

## Materials and Methods

This was a descriptive study carried out at a tertiary care hospital after clearance from an institutional ethics review board. The proximal femur morphology was studied in two groups: Group 1 was called the dry bone group; while Group 2 was labelled as the clinical group.

Group 1 consisted of 50 unpaired dry femur bones from adult human cadavers which were differentiated by the right or left side (26, right; 24,left). Standardised radiographs of the bones in the antero-posterior and lateral views were obtained using the technique described by Rubin *et al*^6,14^. The AP view was obtained by placing the femur in neutral rotation over the cassette. The distance between the radiograph source and the film was 1.2m with the beam centring over the lesser trochanter. For the lateral view, the radiograph source was rotated through 90° in the vertical plane with the distance between the source and the film remaining the same.

**Fig. 1:**
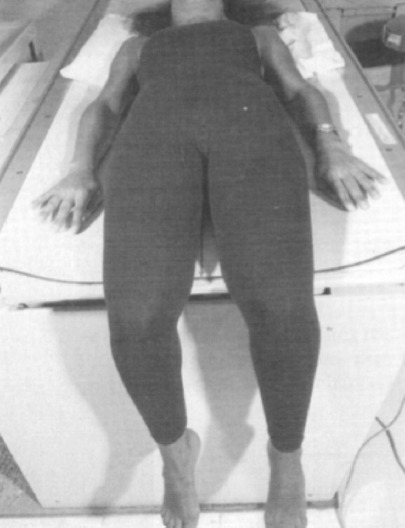
For the AP view the patient was positioned supine on the table with the knees flexed over the edge and the lower portion of the legs perpendicular to the floor.

**Fig. 2:**
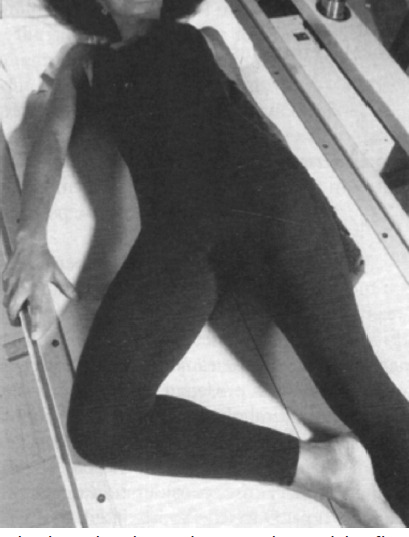
The lateral radiograph were obtained by flexing the index knee, with both the knee and ankle being horizontal to the floor and touching the table top.

The clinical group comprised of 50 adult patients (41 males; 9 females) who presented to the out-patient department. They were in the age group of 40-90 years, without any prior history of long term disease or hospitalisation. Radiographs for the clinical group were done by placing the cassette directly beneath the thigh to reduce magnification (5%). For the AP view, the patient was positioned supine on the table with the knees flexed over the edge and the lower portion of the legs perpendicular to the floor (Fig. 1). Engh *et al* described this as the ideal patient positioning since it produced a standard femoral rotation, and the AP view showed the femur in the true coronal plane^15^. The lateral radiographs were obtained by flexing the index knee, with both the knee and ankle being horizontal to the floor and touching the tabletop (Fig. 2). This ensured that the femur also touched the tabletop and that the radiograph film was taken at right angle to the previously described AP projection.

**Fig. 3:**
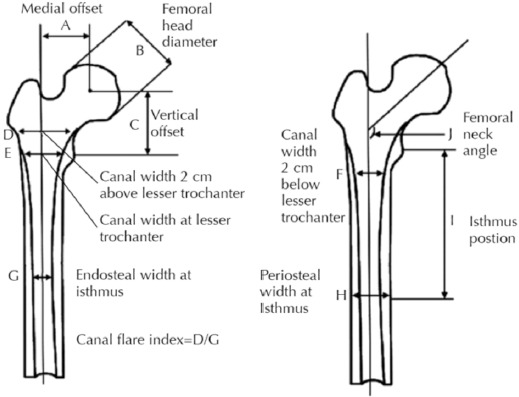
Standard periosteal (extra-cortical) and endosteal (intra-cortical) dimensions in the antero-posterior view.

**Fig. 4:**
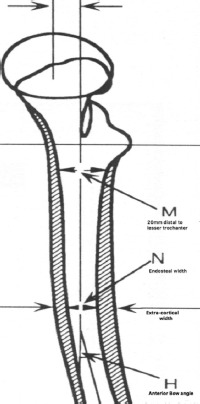
Standard periosteal (extra-cortical) and endosteal (intra-cortical) dimensions in the lateral view.

Bones with visible osseous pathologies such as tumours, deformities, fractures and internal fixation were excluded from the study. Standard periosteal (extra-cortical) and endosteal (intra-cortical) dimensions were determined for each femur according to the method described by Noble *et al*^7^ and are illustrated in Fig. 3 and 4. The extra-cortical measurements were made of the femoral head offset, femoral head diameter, femoral head height, diaphyseal width (medio-lateral), and diaphyseal width (antero-posterior). The intra-cortical measurements were the isthmus position, anterior bow angle and canal width in the medio-lateral and antero-posterior plane (20mm proximal to LT, at LT and 20mm distal to LT).

The isthmus was defined as the narrowest point between the endosteal surface of the medial and lateral cortices. The medullary axis was defined by a line passing through the midpoints of the medullary canal at 20mm proximal and distal to the canal isthmus. The transverse axis was defined by the line that passed through the geometric centre of the lesser trochanter perpendicular to the medullary axis. The height of the femoral head was defined as the distance from the centre of the lesser trochanter to the centre of the femoral head measured parallel to the femoral axis. The axis of the femoral neck was defined as a line passing through the centre of the femoral head that bisected the superior and inferior borders of the femoral neck. The anterior bow angle was calculated as the difference between the axis of the proximal and distal medullary canal as measured on lateral radiograph.

Based on the measurements derived from the AP and lateral radiographs, dimensionless ratios were calculated to express the shape of the proximal femur. The canal flare index was defined and calculated as the ratio of the width of the femur measured 20mm proximal to the lesser trochanter to the width of the medullary canal at the isthmus.

A statistical package SPSS version 21 and MS excel 2018 was used for analysing the collected data. The analysis was done using descriptive statistics wherein the mean and the standard deviation were calculated. Normally distributed continuous variables were tested for significance using the student's t-test. Analysis of variance (ANOVA) was used for comparability between the two groups, and Pearson's correlation coefficient was used to analyse the relationship between the variables. A p-value <0.05 was considered significant.

## Results

The mean value and standard deviation for each measured parameter on the antero-posterior and lateral radiograph for the two groups and subcategories are given in Table I and II. On comparing the two groups, significant differences were found in the femoral head diameter as well as the extra-cortical diaphyseal width in the medio-lateral and antero-posterior planes. Differences could also be noted when comparing intra-cortical canal width proximal, at and distal to the lesser trochanter (LT) in the medio-lateral and antero-posterior planes. The side of the femur bone was not found to significantly influence the measurements in either group. The femoral offset, femoral head height and femoral head diameter were found to be significantly less in females as compared to males, and the average head diameter was 4mm smaller than males. Similarly, the extra-cortical diaphyseal width in the M-L and the intra-cortical canal width in the AP plane were 1.8mm and 2.3mm less than their male counterparts, respectively.

**Table I:** The extra-cortical and intra-cortical radiographic measurements for Group 1 (Dry bone group)

Parameter	Dry bone group (n=50)	Right side (n=26)	Left side (n=24)
Femoral offset	33.98 ± 5.12	32.96 ± 4.93	35.08 ± 5.21
Femoral head height	31.32 ± 6.73	31.00 ± 5.79	31.67 ± 7.73
Femoral head diameter	44.80 ± 4.20	44.46 ± 3.97	45.16 ± 4.48
Diaphyseal width(M-L)	25.26 ± 2.38	24.69 ± 2.31	25.87 ± 2.35
Diaphyseal width(A-P)	24.48 ± 2.26	24.42 ± 2.00	24.54 ± 2.55
Isthmus position	107.38 ± 8.81	107.23 ± 8.28	107.54 ± 9.53
Neck shaft angle	137.80 ± 6.90	137.27 ± 7.23	138.38 ± 6.65
Anterior bow angle	8.38 ± 2.75	8.42 ± 2.69	8.33 ± 2.88
Canal width(M-L) 20mm proximal to LT	40.42 ± 3.84	39.92 ± 4.44	40.95 ± 3.08
Canal width(M-L) at LT	23.16 ± 3.20	22.69 ± 3.18	23.67 ± 3.21
Canal width(M-L) 20mm distal to LT	16.26 ± 2.23	16.03 ± 2.39	16.50 ± 2.06
Canal width(M-L) at isthmus	11.00 ± 1.90	11.31 ± 2.28	10.67 ± 1.38
Canal width(A-P) 20mm distal to LT	16.90 ± 2.27	16.88 ± 2.39	16.91 ± 2.18
Canal width(A-P) at isthmus	14.18 ± 2.08	14.04 ± 1.75	14.34 ± 2.44

**Table II:** The extra-cortical and intra-cortical radiographic measurements for Group 2 (Clinical group)

Parameter	Clinical group (n=50)	Right side (n=26)	Left side (n=24)	Male (n=41)	Female (n=9)
Femoral offset	34.92 ± 6.76	35.00 ± 6.94	34.83 ± 6.72	35.92 ± 6.84	30.33 ± 4.18
Femoral head height	31.44 ± 6.30	30.84 ± 6.35	32.08 ± 6.31	32.24 ± 6.27	27.78 ± 5.29
Femoral head diameter	53.04 ± 4.26	53.23 ± 4.35	52.83 ± 4.25	27.12 ± 1.55	23.78 ± 2.33
Diaphyseal width (M-L)	28.04 ± 2.28	28.15 ± 2.18	27.91 ± 2.41	28.36 ± 2.89	26.56 ± 1.59
Diaphyseal width (A-P)	27.18 ± 2.76	28.15 ± 2.18	27.91 ± 2.41	27.31 ± 2.76	26.56 ± 2.88
Isthmus position	110.58 ± 10.37	108.62 ± 9.38	112.71 ± 11.15	110.10 ± 9.91	112.78 ± 12.71
Neck shaft angle	138.72 ± 6.43	138.38 ± 5.66	139.08 ± 7.27	139.10 ± 6.82	137.11 ± 4.14
Anterior bow angle	8.48 ± 2.26	8.34 ± 1.64	8.62 ± 2.81	8.58 ± 2.33	8.00 ± 1.93
Canal width (M-L) 20mm proximal to LT	43.36 ± 4.62	43.23 ± 4.81	43.50 ± 4.51	43.51 ± 4.77	42.67 ± 4.06
Canal width (M-L) at LT	25.62 ± 3.40	25.96 ± 3.34	25.25 ± 3.50	25.46 ± 3.10	26.33 ± 4.69
Canal width (M-L) 20mm distal to LT	19.18 ± 2.86	19.34 ± 2.94	19.00 ± 2.83	19.12 ± 2.81	19.44 ± 3.24
Canal width (M-L) at isthmus	11.47 ± 2.35	11.25 ± 2.61	11.70 ± 2.05	11.25 ± 2.31	12.44 ± 2.4
Canal width (A-P) 20mm distal to LT	18.78 ± 3.71	19.26 ± 4.22	18.25 ± 3.08	18.92 ± 3.78	18.11 ± 3.55
Canal width (A-P) at isthmus	13.85 ± 2.68	13.69 ± 2.87	14.02 ± 2.50	13.43 ± 2.44	15.72 ± 3.05

In the clinical group, we analysed radiographic parameters in different age categories with a 10 years interval over the age range 40 - 89 years. ANOVA was used to compare the difference within groups which revealed a significant change only in the intra-cortical canal width measurements in both ML and AP at different landmarks as against other parameters.

Pairwise correlation of the different morphometric parameters in both groups, which were found to be statistically associated, is outlined in (Table III and IV). Though most significant associations had strong correlations (r > 0.5), not all parameters were uniformly correlated across both groups.

**Table III:** Typical values of correlation co-efficient for pairwise correlation of femoral dimensions for Group 1

Variables	Correlation coefficient (r)	Statistical significance (p)
**Periosteal/periosteal**		
Head position vs femoral head diameter	0.629	P < 0.0001
Head position vs neck shaft angle	0.762	P < 0.0001
Femoral head diameter vs Diaphyseal width M-L	0.535	P < 0.0001
Femoral offset vs diaphyseal width M-L	0.589	P < 0.0001
**Endosteal/endosteal**		
Proximal to LT vs At LT	0.601	P < 0.0001
Proximal to LT vs Distal to LT	0.480	P < 0.0001
At LT vs Distal to LT	0.731	P < 0.0001
Distal to LT vs At isthmus	0.658	P < 0.0001
Distal to LT vs At isthmus (AP)	0.589	P < 0.0001
At isthmus vs At isthmus (AP)	0.638	P < 0.0001
Distal to LT (AP) vs At isthmus (AP)	0.500	P < 0.0001

**Table IV:** Typical values of correlation co-efficient for pairwise correlation of femoral dimensions for Group 2

Variables	Correlation coefficient (r)	Statistical significance (p)
**Periosteal/periosteal**		
Femoral head diameter vs diaphyseal width M-L	0.576	P < 0.0001
Femoral offset vs neck shaft angle	0.511	P < 0.0001
Diaphyseal width M-L vs anterior bow angle	0.524	P < 0.0001
**Endosteal/endosteal**		
Proximal to LT vs At LT	0.638	P < 0.0001
Proximal to LT vs Distal to LT	0.513	P < 0.0001
Proximal to LT vs Distal to LT (AP)	0.494	P < 0.0001
At LT vs Distal to LT	0.698	P < 0.0001
At LT vs Distal to LT (AP)	0.677	P < 0.0001
Distal to LT vs At isthmus	0.500	P < 0.0001
Distal to LT vs At isthmus (AP)	0.634	P < 0.0001
Distal to LT vs Distal to LT (AP)	0.695	P < 0.0001
At isthmus vs At isthmus (AP)	0.749	P < 0.0001

**Fig. 5:**
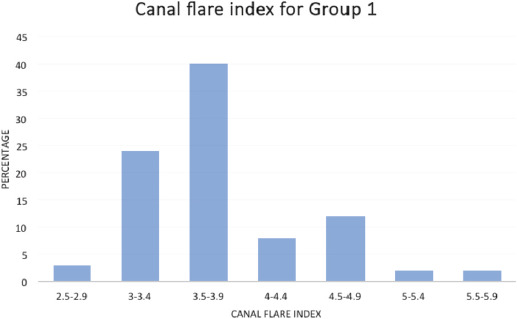
Canal flare index for Group 1.

**Fig. 6:**
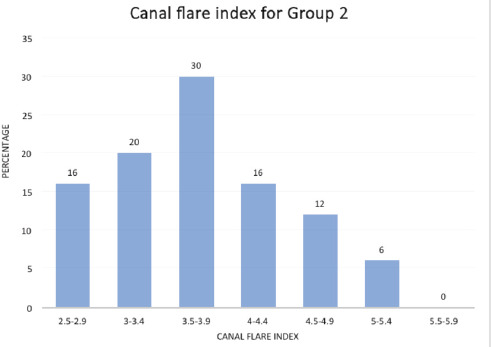
Canal flare index for Group 2.

The canal flare index (CFI) helps to categorise the femur into three types based on the shape viz. normal, stovepipe and champagne flute. Canal flare indices less than 3 indicate stovepipe, 3-4.75 imply normal, and more than 4.75 are canals with champagne flute appearance. The CFI for the two groups is illustrated in Fig. 5 and 6. Over the anatomic range, the CFI (defined as the ratio of the width of the femur measured 20mm proximal to the lesser trochanter to the width of the medullary canal at the isthmus) was broadly distributed from 2.5 to 5.75. In our study, the CFI was found to be distributed in a skewed normal distribution with maximum incidence at 3.5. However, a substantial number of bones had incidences between 3.25 and 4.75.

## Discussion

The purpose of our study was to gain further insight into geographical variation in bone morphology in a south Indian population. Numerous studies have previously highlighted the importance of a close match in the dimensions of the proximal femur and the implanted prosthesis to achieve optimal results^4-11^. Indian proximal femoral morphology grossly differs when compared to western literature^13^. The plausibility of such a disparity is not surprising since India is an ethnically and culturally diverse nation with varying practices within the indigenous population.

We studied the proximal femur morphology in a south Indian population in two distinct groups. In our study, the femoral head offset and femoral head diameter was 5.6mm and 5mm more in males, respectively. Similarly, the diaphyseal width also showed an increase with respect to their female counterparts. However, no significant difference was noted between extra-cortical and intra-cortical parameters based on the sides. Canals of the older femur in medio-lateral plane and antero-posterior plane showed increased width compared to younger age groups. It was also observed that a significant difference existed in measurements made on radiographs obtained from a cadaveric bone as compared to the living patients.

The comparison of our results for the dry bone and the clinical group in contrast to other studies within India and worldwide is illustrated in Table V. A significant difference was noted in the morphometric parameters across various population subsets within the Indian subcontinent, as well as with the Western population, suggestive of regional variation. The femoral canal lacks uniformity with regard to its shape and assumes a broad morphological variety, ranging from a stovepipe to a champagne flute. The broad range of the canal flare index suggests a poor correlation between distal and proximal dimensions of the medullary canal. Thus, to optimise metaphyseal load transfer in THA, the femoral components must be selected based on the proximal canal fit and not the diameter of the isthmus. The average neck shaft angle in the Indian femora is around 128° with higher values reported in southern India^16^. We observed a higher neck shaft angle in both groups, which were in contrast to that noted by Siwach *et al*^13^. Our findings, however, were comparable to those reported by Sengodan *et al*^17^, Deshmukh *et al*^18^ and Saikia *et al*^19^ wherein they reported higher neck shaft angles for the south, west and north-eastern Indian population, respectively. Anderson *et al*^20^ studied inter-populational variation in human femoral neck shaft angles across 30 modern, historic and prehistoric human population samples and concluded that urban population have a higher neck shaft angle as compared to the non-mechanised rural population. A consistent pattern of an increase in angle with an increase in the incidence of a sedentary lifestyle has been observed. The findings in our study may be attributed to the variable ethnicity, urban demographic profile and a high human development index of our study population^21^.

**Table V:** Comparison of proximal femur morphology with previous studies in the Indian sub-continent, Thai and Western population

	Present study dry bone group (N =50)	Present study clinical group (N =50)	Siwach *et al*^13^ (Indian; N=75)	Umer *et al*^23^ Pakistan; N=136; healthy volunteers)	Noble *et al*^7^ (Western; N=200)	Rubin *et al*^6^ (Western; N=32)	Thai^12^
Femoral head offset	33.9 ± 5.12	34.92 ± 6.76	38	41.9	43	47	-
Femoral head height	31.3 ± 6.72	31.44 ± 6.30	50.15	56.0	51.6	56.1	48.94
Femoral head diameter	44.8 ± 4.19	53.04 ± 4.26	43.53	50.1	46.1	43.4	43.98
Diaphyseal width M-L	25.2 ± 2.38	28.04 ± 2.28	24.42	27.9	27.0	26.7	27.21
Diaphyseal width A-P	24.48 ± 2.26	27.18 ± 2.76	-	-	-	-	-
Isthmus position	107.3 ± 8.81	110.58 ± 10.37	112.9	105.7	113.4	105.7	112.93
Neck shaft angle	137 ± 6.90	138.72 ± 6.43	123	130.3	124.7	122.9	128.04
Anterior bow angle	8.38 ± 2.75	8.48 ± 2.26	-	-	-	-	5.75
Canal width M-L proximal to LT	40.4 ± 3.84	43.36 ± 4.62	43.5	47.4	45.4	43.1	-
Canal width at LT	23.16 ± 3.20	25.62 ± 3.40	23.8	28.3	29.4	27.9	-
Canal width Distal to LT	16.2 ± 2.22	19.18 ± 2.86	16.57	21.1	20.9	21.0	-
Canal width at isthmus	11 ± 1.90	11.47 ± 2.35	10.11	11.0	12.3	13.1	10.05
Canal flare index	3.7 ± 0.6	3.8 ± 0.7	-	4.47	3.8	3.36	-

Despite the wide regional variation, hip arthroplasty prosthesis is not manufactured based on region, country or race. Mahaisavariya *et al*^12^ conducted a study on 108 dry femurs to prove that the femoral head diameter was smaller and the average anteversion angle was higher than that of standard Caucasian size. To improve the outcome and prevent avoidable complications, region or race specific implants are the need of the hour. Uncemented arthroplasty gives good press fit and bone growth, thereby increasing early stability for full weight-bearing. Rawal *et al*^22^ found a difference of 16.8% in femoral head offset between Indian and Swiss population, which in turn can alter the soft tissue tension and range of movements. Measurements taken 20mm above the lesser trochanter showed a difference of 45.4% when compared with the French population, which in turn plays a major role in mechanical stability. Therefore, we must increase our understanding of the morphology of the proximal femur and work towards developing the prosthesis that can provide the best outcome with the least complications for the patient.

The unique aspect of this study was that the proximal femur morphology was assessed in both cadaveric bones and healthy volunteers and the two groups were compared. In addition, we illustrated a comparison between various ethnicities. Certain additional parameters such as anterior bow angle and canal flare index for an Indian population were also measured. Though conventional radiography is the most cost effective method available, its accuracy is less compared to computed tomography. All measurements were taken over templates on the radiographs and variations due to magnification were a shortcoming of this study. Furthermore, we were unable to differentiate the cadaveric bones based on sex.

## Conclusion

Morphologically, the proximal femur shows wide geographic variation, and it is crucial that hip arthroplasty prosthesis designs are modified and tailor made for optimal results. South Indian population needs specifically customised implants with increased neck shaft angle and decreased intra as well as extra-cortical width for press fit in hip arthroplasty. Measurements made on cadaveric bones significantly differ from those in live patients. The variation between the two sexes must also be accounted for.

## References

[1] Daigle ME, Weinstein AM, Katz JN, Losina E (2012). The cost-effectiveness of total joint arthroplasty: a systematic review of published literature. Best Pract Res Clin Rheumatol..

[2] Bayliss LE, Culliford D, Monk AP, Glyn-Jones S, Prieto-Alhambra D, Judge A (2017). The effect of patient age at intervention on risk of implant revision after total replacement of the hip or knee: a population-based cohort study. Lancet..

[3] Evans JT, Evans JP, Walker RW, Blom AW, Whitehouse MR, Sayers A (2019). How long does a hip replacement last? A systematic review and meta-analysis of case series and national registry reports with more than 15 years of follow-up. Lancet..

[4] Dorr LD, Luckett M, Conaty JP (1990). Total hip arthroplasties in patients younger than 45 years. A nine- to ten-year follow-up study. Clin Orthop Relat Res..

[5] Poss RP, Walker P, Spector M, Reilly DT, Robertson DD, Sledge CB (1988). Strategies for improving fixation of femoral components in total hip Arthroplasty. Clin Orthop Relat Res..

[6] Rubin PJ, Leyvraz PF, Aubaniac JM, Argenson JN, Estève P, de Roguin B (1992). The morphology of proximal femur. A three-dimensional radiographic analysis. J Bone Joint Surg Br..

[7] Noble PC, Alexander JW, Lindahl LJ, Yew DT, Granberry WM, Tullos HS (1988). The anatomic basis of femoral component design. Clin Orthop Relat Res..

[8] Stiehl JB, Jacobson D, Carrera G (2007). Morphological analysis of the proximal femur using quantitative computed tomography. Int Orthop..

[9] Krishnamurthy AB, MacDonald SJ, Paprosky WG (1997). 5- to 13-year follow-up study on cementless femoral components in revision surgery. J Arthroplasty..

[10] Walker PS, Douglas D, Robertson DD (1988). Design and fabrication of cementless hip stems. Clin Orthop Relat Res..

[11] Noble PC, Box GG, Kamaric E, Fink MJ, Alexander JW, Tullos HS (1995). The effect of aging on the shape of the proximal femur. Clin Orthop Relat Res..

[12] Mahaisavariya B, Sitthiseripratip K, Tongdee T, Bohez ELJ, Sloten JV, Oris P (2002). Morphological study of the proximal femur: a new method of geometrical assessment using 3-dimensional reverse engineering. Med Eng Phys..

[13] Siwach RC, Dahiya S (2003). Anthropometric study of proximal femur geometry and its clinical application. Indian J Orthop..

[14] Rubin PJ, Leyvraz PF, Heegaard JH (1989). Radiologic changes of anatomic parameters of the proximal femur as a function of its position in rotation. Rev Chir Orthop Reparatrice Appar Mot..

[15] Engh CA, Bobyn JD, Glassman AH (1987). Porous-coated hip replacement. The factors governing bone ingrowth, stress shielding, and clinical results. J Bone Joint Surg Br..

[16] Kate BR, Robert SL (1963). The angle of femoral torsion. J Anat Soc India..

[17] Sengodan VC, Sinmayanantham E, Kumar JS (2017). Anthropometric analysis of the hip joint in South Indian population using computed tomography. Indian J Orthop..

[18] Deshmukh TR, Kuthe AM, Ingole DS, Thakre SB (2010). Prediction of femur bone geometry using anthropometric data of Indian population: a numerical approach. J Med Sci..

[19] Saikia KC, Bhuyan SK, Rongphar R (2008). Anthropometric study of the hip joint in Northeastern region population with computed tomography scan. Indian J Orthop..

[20] Anderson JY, Trinkaus E (1998). Patterns of sexual, bilateral and interpopulational variation in human femoral neck-shaft angles. J Anat..

[21] United Nations Development Programme (UNDP) (2019). Human Development Report 2019 Beyond income, beyond averages, beyond today: Inequalities in human development in the 21st century..

[22] Rawal BR, Ribeiro R, Malhotra R, Bhatnagar N (2012). Anthropometric measurements to design best-fit femoral stem for the Indian population. Indian J Orthop..

[23] Umer M, Sepah YJ, Khan A, Wazir A, Ahmed M, Jawad MU (2010). Morphology of the proximal femur in a Pakistani population. J Orthop Surg (Hong Kong)..

